# Mutual Information of Multiple Rhythms for EEG Signals

**DOI:** 10.3389/fnins.2020.574796

**Published:** 2020-12-14

**Authors:** Antonio José Ibáñez-Molina, María Felipa Soriano, Sergio Iglesias-Parro

**Affiliations:** ^1^Department of Psychology, University of Jaén, Jaén, Spain; ^2^Unidad de salud mental, Hospital San Agustín, Linares, Spain

**Keywords:** cross-frequency coupling, mutual information, EEG rhythms, multiscale interactions, neural oscillations

## Abstract

Electroencephalograms (EEG) are one of the most commonly used measures to study brain functioning at a macroscopic level. The structure of the EEG time series is composed of many neural rhythms interacting at different spatiotemporal scales. This interaction is often named as cross frequency coupling, and consists of transient couplings between various parameters of different rhythms. This coupling has been hypothesized to be a basic mechanism involved in cognitive functions. There are several methods to measure cross frequency coupling between two rhythms but no single method has been selected as the gold standard. Current methods only serve to explore two rhythms at a time, are computationally demanding, and impose assumptions about the nature of the signal. Here we present a new approach based on Information Theory in which we can characterize the interaction of more than two rhythms in a given EEG time series. It estimates the mutual information of multiple rhythms (MIMR) extracted from the original signal. We tested this measure using simulated and real empirical data. We simulated signals composed of three frequencies and background noise. When the coupling between each frequency component was manipulated, we found a significant variation in the MIMR. In addition, we found that MIMR was sensitive to real EEG time series collected with open vs. closed eyes, and intra-cortical recordings from epileptic and non-epileptic signals registered at different regions of the brain. MIMR is presented as a tool to explore multiple rhythms, easy to compute and without *a priori* assumptions.

## Introduction

Rhythmical activity in the waking brain is organized in space and time at different scales. It is widely accepted that coupling at multiple oscillatory frequencies allows flexible coordination between neural assemblies (e.g., Canolty and Knight, 2010). This multiscale nature of neural circuits is captured in the structure of neurophysiological measures as EEGs. The EEG consists of scalp recordings of voltage variations in microvolts from large cortical areas, with frequencies traditionally ranging from 0.1 to about 100 Hz. It is noteworthy that the relationship between voltage amplitude and frequency follows a 1/*f*^α^ distribution, which means that the relationship between the frequency of oscillations and the amplitude of the signal follows an inverse power law (Buzsáki et al., 2013). Therefore, while fast oscillations show small amplitudes, slow ones exhibit large amplitudes (frequencies around 10 Hz are the exception to this rule). An important feature of these oscillations is that different frequencies interact to facilitate cortical communication and integration (Fell and Axmacher, 2011; Lisman and Jensen, 2013; Knyazev et al., 2019). This phenomenon is called cross frequency coupling (CFC) and can be empirically explored with the study of signals dependencies at different frequencies (note that in this research work, we will use rhythms and frequencies as synonyms).

Experimental work on CFC has been mainly focused on three types of coupling: amplitude-amplitude coupling, phase-phase coupling, and phase-amplitude coupling (PAC), the latter being the one that has generated the most fruitful research. For example, Axmacher et al. (2010) explored the role of theta-gamma PAC in working memory. They presented participants with trials with 2, 3, or 4 pictures of faces that they needed to remember after a short time interval. The intracranial EEG was bilaterally recorded from the hippocampus during the maintenance interval. They found higher theta-gamma PAC during WM retention than in a baseline condition. Furthermore, they showed that the average frequency in theta in the PAC was faster during maintenance of one face as compared to maintenance of four faces. This result suggests that PAC is sensitive to working memory load in a very specific way, namely that hippocampal oscillations in theta range adapt its speed to regulate the gamma amplitude at different memory load conditions.

PAC has been measured in other cognitive domains as sensory signal detection (Handel and Haarmeier, 2009), or attentional selection (Schroeder and Lakatos, 2009), among others. In addition, evidence of this phase-amplitude interaction has been found in other species apart from humans (Axmacher et al., 2010), such as in mice (Wulff et al., 2009) or monkeys (Lakatos et al., 2005), a finding that stresses the relevance of this mechanism for general cognition.

The attempt to measure the PAC has generated different empirical indicators, but the three main measures are: the Mean Vector Length Modulation Index (Canolty et al., 2006), the Kullback-Leibler Modulation Index (Tort et al., 2010), and the General Linear Model Modulation Index (Penny et al., 2008). These indicators have shown promising results in different cognitive domains, such as attention (Szczepanski et al., 2014), perception (Voytek et al., 2010), learning (Tort et al., 2008), or spontaneous activity (Cheng et al., 2016), among others. However, most of these methods have important drawbacks. First, a high number of points is needed in order to provide robust estimates of PAC; in general, the number of points is determined by the frequency of the slowest wave of interest. In addition, since these indicators are sensitive to the noise in the signal, and the amount of noise may vary among different data sets, some authors (Tort et al., 2010) have proposed that the number of cycles needed to compute a reliable PAC estimate may be more than 200. Another caveat is that the researcher must specify *a priori* the frequency bands at which synchronization will be assessed (Cohen, 2008), and when attempts have been made to overcome this limitation, as with the Mean Vector Length Modulation Index (Canolty et al., 2006), the temporal dynamics of cross-frequency coupling have been lost. Most of these methods, moreover, only allow the study of the PAC between two variables as, for example, in the aforementioned studies that address the relationship between theta phase and gamma amplitude, thus excluding more complex interaction patterns, which would involve more than two frequency bands.

In this research work, we propose a new measure, based on a different perspective of CFC, in order to avoid these limitations. Our approach has its roots in previous research works on symbolic time-series analysis. Symbolic analysis is an empirical approach to characterize complex data by discretizing it and obtaining a new data set that represents the underlying dynamics of the generating process. The common feature of this analysis is the transformation of the original data into sequences of a few symbols, followed by the analysis of the structure of the obtained symbolic sequences (Alcaraz, 2018). Although it involves some loss of information, in symbolic analysis the efficiency of numerical computation is greatly increased over the analyses on the original signal (Piccardi, 2004). Symbolic data analysis, on the other hand, appears to be less sensitive to the presence of noise (Cuéllar and Binder, 2001; Graben, 2001; Daw et al., 2003). Although symbolic analysis has had previous applications in the context of EEG signal analysis (see for example, Wendling et al., 1996; Graben et al., 2000; Hively et al., 2000; García-Martínez et al., 2017), to our knowledge this is the first study employing a symbolic analysis on EEG that allows a multi-scale approach.

Recently, Martínez-Cancino et al. (2019) have developed a measure that used mutual information to estimate CFC. In general, they followed the same procedure of classical CFC to obtain phases and amplitudes of the signal. The main difference with previous measures is that they calculated the mutual information of the phase-amplitude as the main indicator of PAC (for further details see Martínez-Cancino et al., 2019). Importantly, mutual information of phase-amplitude is a mathematical estimation of the dependence between the phase of the slow rhythm and the amplitude of the rapid rhythm. These authors showed that this strategy is adequate and can be easily adapted to ERPs experiments, but it maintains the main limitations of classical CFC mentioned above.

Our proposal in this work, for the analysis of the symbolic sequences, is also based on mutual information (for a complete review on the application of information theory in the field of Neuroscience we recommend the excellent work of Timme and Lapish, 2018). However, the rationale is quite different because we obtained CFC without any calculation of phase or amplitude at a particular frequency band. The main idea is to obtain series of binary sequences based on the original EEG series, in such a way that each of them includes information from a particular rhythm or oscillatory activity. We then combine the information from all the series into a new symbolic series. Later, we calculated the delayed mutual information of these symbolic sequences as an estimation of dependence between them. For example, if we aim to investigate the interaction between the theta, beta and gamma oscillations, we could first obtain one binary series for each frequency band; second, we would obtain a symbolic series, constructed with integers, that collected for each time step the information contained in the binary series; and finally, we would calculate the mutual delayed information of this symbolic series to estimate the dependence between the three rhythms. If the activity from these three rhythms is independent, the delayed mutual information would be low; but if the rhythms are coupled, our estimator would be high. Because the mutual information covers the relations across all frequencies implicitly, the solution space is reduced compared to CFC based techniques.

Hence, the proposed measure is an estimator of the dependence between activity at *n* different oscillatory rhythms or scales. We called this measure MIMR, an acronym of Mutual Information of Multiple Rhythms. The main advantage of this approach is that it is possible to explore the coupling of more than two rhythms for a given function. We will describe the measure in detail in the next section.

## Materials and Methods

### Mutual Information of Multiscale Rhythms

**FIGURE 1:**
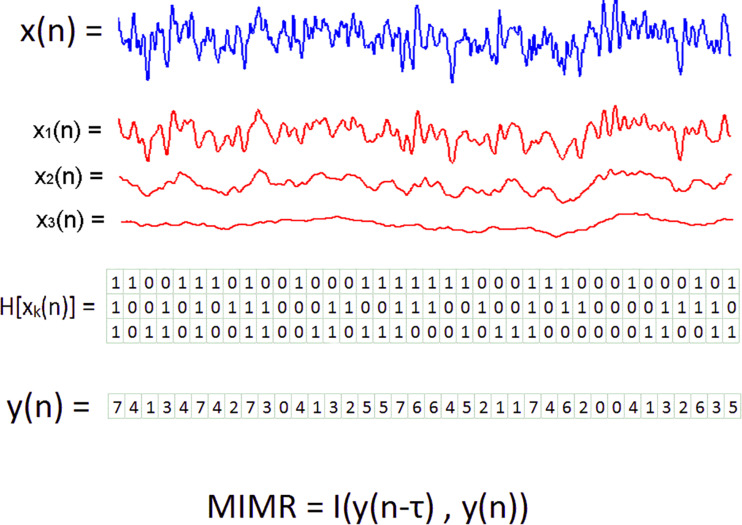
Graphical description of the method to obtain MIMR from three scales or rhythms. In the first step, the original time series, in blue, are transformed into multiple binary series. Binary series are represented in a matrix in which the rows are the series at each scale and the columns represent the state of all scales at a given time step. To construct these new binary series, smoothed versions of the signal, here in red, are subtracted one to each other. In a second step, each column of the matrix is considered a single integer and the *Y*(n) series is created. The last step consists of the calculation of time delayed mutual information to *Y*(n).

From the original signal, we obtained multiple binary sequences capturing different time scales. These binary sequences were obtained using smoothed versions of the original signal as thresholds. The smoothed procedure was implemented with median moving windows of different length. The process goes as follows: First, the original signal was smoothed (filtered) using different window sizes. The resulting signals would show more rapid activity (high frequency) depending on the size of the window. Second, a binary sequence on a particular scale was obtained by subtracting all data points from two smoothed versions using successive window sizes. Negative values in the subtraction were assigned to 0 and positive values to 1. For example, if the original series are smoothed using a window of 201 points, and these values are compared with another smoothed version with a window of 101 points, the obtained binary sequence would reflect the differential activity between both scales. It is also possible to relate the window size with a particular frequency by knowing the AD rate of the signal. For example, for an AD rate of 1,000 Hz a window size of 201 would correspond with a frequency of 1,000/201 ^~^5 Hz and a window size of 101 would be more sensitive to 1,000/101 ^~^10 Hz. In this particular example, the binary sequence obtained with the subtraction of both smoothed signals would contain the activity between 5 and 10 Hz. Once we have built the set of binary sequences with the same length, we transform them into a single signal. This new signal is a symbolic sequence composed of integers, each of them representing the binary values at each scale. Each integer in the sequence was assigned by taking together all binary values at that time step as a single integer in a base of 2. Then, for simplicity, these binary numbers were transformed into integers in a base of 10 (see Figure 1 for a graphical description of the entire procedure). For example, if we obtained three binary sequences, and in time step 1 the values of each one were 1, 0 and 1; we took the number 101 as a number in a base of 2, and transformed it to 5 in a base of 10.

Formally, each signal {*x*(n)} was smoothed using a median moving window with *m* window sizes {*w_*k*_*,…, *w_*m*_*} for *k* = *1,…,m*. We obtained the binary sequences using the following expression

(1) $$\begin{array}{c} H_{k}\left[x_{k}\left(n\right)\right]=H[median(x_{k-1}\left(n-\left(w_{k-1}-1\right)/2\right),\ldots ,x_{k-1}\left(n\right),\\ \;\ldots ,x_{k-1}\left(n+\left(w_{k-1}-1\right)/2\right))-median(x_{k}\left(n-\left(w_{k}-1\right)/2\right),\\ \;\ldots ,x_{k}\left(n\right),\ldots ,x_{k}\left(n+\left(w_{k}-1\right)/2\right))] \end{array}$$

where (*w*_*m*_−1)/2 are the total points from the sides that are not included in the smoothing; *w*_0_ corresponds to the original signal *x*(n); and H[n] is the discrete Heaviside function:

(2) $$H\left[n\right]=\left\{ \begin{array}{c} 1ifn>1\\ 0ifn\leq 0 \end{array}\right.$$

All binary sequences were made with the same length (*N-w_*m*_ -1*); and therefore, for each time point we had a vector with *m* binary values. Each of these vectors were transformed into an integer so we could have single values to identify all possible multiscale states. With these values we constructed the new signal {*Y(n)*} in which we assigned numbers by considering all values in the vectors as a single number in a base of 2. After that, we simply changed this number to a base of 10. Hence, the number of possible integers in {*Y(n)*} was 2^*m*^ (see Figure 1).

Finally, the MIMR was obtained as the delayed mutual information of integers in {*Y(n)*} using the delayed mutual information:

(3) $$\begin{array}{c} MIMR=I\left(Y\left(n-\tau \right),Y\left(n\right)\right)=\\ \sum_{n}P\left(Y\left(n-\tau \right),Y\left(n\right)\right)log_{2}\frac{P\left(Y\left(n-\tau \right),Y\left(n\right)\right)}{P\left(Y\left(n-\tau \right)\right)P\left(Y\left(n\right)\right)} \end{array}$$

which is a measure that gives the average bits predicted in *Y(n)* given the state *Y(n-*τ*).* It evaluates, therefore, the linear and non-linear dependencies between two time series, so it has been used to quantify the linear and non-linear statistical coupling between biomedical signals (Escudero et al., 2009). The algorithm to obtain mutual information needs the calculation of probabilities and joint probabilities at all estates *Y(n)* and *Y(n-*τ*)*. The parameter τ can be estimated using the first value that is closer to zero in the autocorrelation function of *Y(n).*

### Validation of MIMR With Signal Simulations

In order to validate the new measure it was necessary to demonstrate that it is sensitive to the degree of coupling between rhythms at different frequencies, which are part of the signal. To this end, we designed two different sets of simulations. In the first set, we manipulated the coupling between added wavelets in a signal. In the second set, we manipulated the PAC of two sinusoidal signals at 8 and 60 Hz. The data corresponding to these synthetic signals are stored in Synapse, the repository of Sage bionetworks, with identification number syn22914762. The data are available on request.

#### Simulations Based on Wavelets

We constructed signals by means of the addition of three wavelets at different oscillatory frequencies. The coupling between the wavelets was manipulated by changing the degree of time locking between them. Each signal, then, was constructed using a base of 300 wavelets distributed along the signal. There were 100 wavelets per rhythm or frequency. The coupling was manipulated with the variability in time coupling between the wavelets at different frequencies. When these wavelets were time locked, we would expect high values of MIMR. However, if wavelets at different frequencies were distributed using a random delay, MIMR was expected to be lower.

**FIGURE 2:**
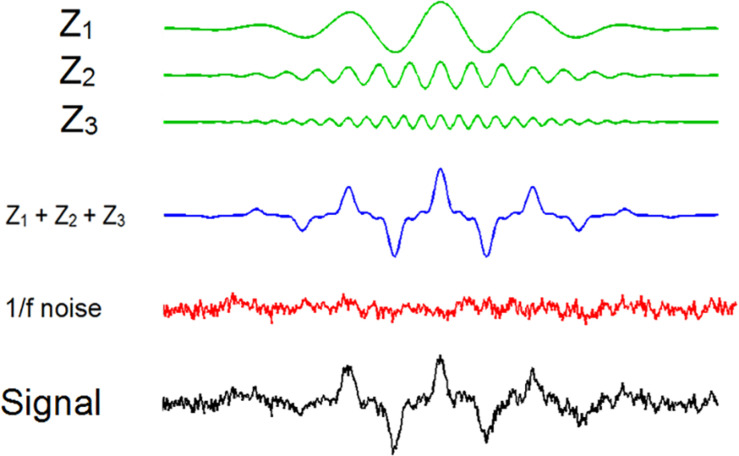
Graphical description of the process to obtain the signals with three wavelets and 1/f noise. Note that, for simplicity, in this picture we included only a single wavelet.

The signal *x*(t) was constructed using three frequencies ×100 wavelets of the type (see Figure 2 for a graphical description of the signal)

(4) $$z_{n}\left(t\right)=A_{n}\cdot e^{-t^{2}/2}\cdot cos\left(2\pi f_{n}t\right),$$

where *A*_*n*_ and *f*_*n*_ are the amplitude and frequency of the wavelets at frequency n. The separation in points between each *z*_*n*_(*t*)at the same frequency *n* was calculated with a random number between 100 and 190 points. Time locking between *z*_*n*_*s*at different frequencies was manipulated using random numbers between 1 and 90 points. Hence, we included random delays across wavelets at the same frequency and wavelets at different frequencies being the former the key parameter to manipulate time locking. The parameters we used for the wavelets were: *A*_1_ = 4; *A*_2_ = 2; *A*_3_ = 1; *f*_1_ = 1; *f*_2_ = 3; *f*_3_ = 5; *time step* = 0.01; *wavelet duration* = 601 *points*; Finally, we added noise to the entire signal. We selected a noise with 1/*f* power distribution (pink noise) which produces a EEG-like signal (Novikov et al., 1997).

**FIGURE 3:**
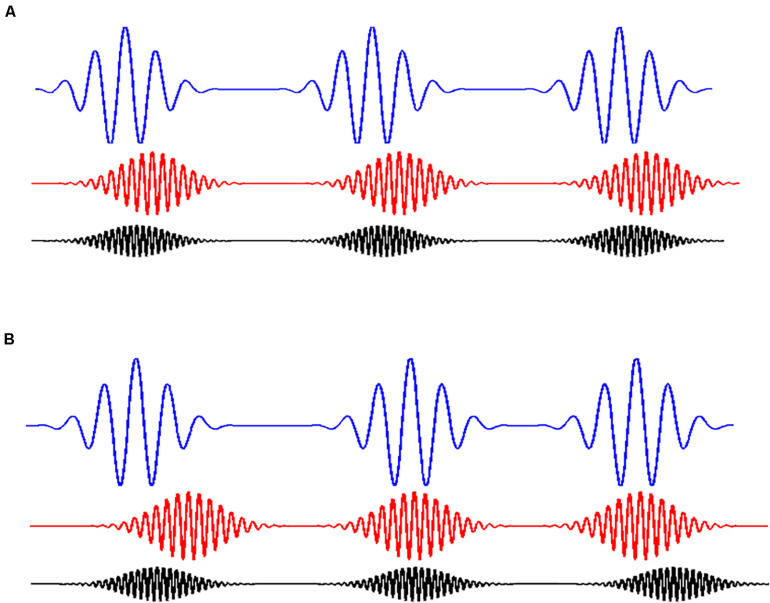
Coupling of rhythms. **(A)** Shows three coupled rhythms. They are coupled because the wavelets at different frequencies (in different colors) are time locked between them. On the contrary. **(B)** Shows the case in which the wavelets at different frequencies are not coupled. In this case, it can be observed that the appearance of a wavelet at a particular time is independent of the appearance of the other two wavelets.

In our study we manipulated the level of noise in the signal and the degree of coupling between three frequencies (see Figure 3). The mean signal-to-noise ratios used in the simulations were 1.8, 7.6, and 18.2 dB. The goal was to assess to what extent our measure was sensitive to frequencies with different couplings in the presence of added noise. To obtain the binary sequences, we used windows sizes of (WS: 21, 33, 103) and a time delay parameter τ with a value of 40.

#### Simulations Based on PAC

Another set of simulations were constructed to test if MIMR could capture PAC between different frequencies in a signal. We simply added two sinusoidal signals at *f*_1_ = 8 Hz, and *f*_2_ = 60 Hz and modulated the amplitude of the rapid wave with the phase of the slow one. As in previous simulations, we added 1/f with signal-to-noise-ratio of 1.8 dB. In this case, the time step was set to 0.001. The signal was generated as

(5) $$x\left(t\right)=bcos\left(2\pi f_{1}t+\zeta \right)+A\left(t\right)\text{cos}\left(2\pi f_{2}t\right)$$

where *A*(*t*) = cos (2π*f*_1_*t*) + 1 is the amplitude of the sinusoidal component with *f*_2_.

The parameter b was introduced to increase the amplitude of the slow frequency *f*_1_ with respect to the fast frequency *f*_2_. This parameter b was set to 4 in our simulations.

We manipulated PAC by introducing ζ as a random value in the phase of the slow signal. In the PAC condition, ζ was set to zero, but in the No-PAC condition, ζ ranged on the interval ± 0.5. Because ζ does not have an effect on the amplitude of the fast component, PAC would be lower when ζ ≠ 0, and we expected lower values of MIMR as well. Window lengths used in the moving medians for the calculation of MIMR were (WS: 19, 127) and the time delay parameter τ was set to 40.

### Validation of MIMR With Empirical Data

We also tested MIMR in two sets of empirical data: from patients with epilepsy, and individuals with closed and open eyes. We used data from patients with epilepsy because research has shown that during ictal episodes there is highly synchronized activity in large areas of the cortex. This synchronization reflects transient couplings between neural activity at several frequencies (e.g., Lehnertz et al., 2014), and these couplings should be captured with a measure sensitive to CFC. If MIMR is able to capture changes in frequency coupling, it should vary with signals from patients with epilepsy. Our hypothesis is that intracraneal signals registered during epileptic seizures should exhibit higher MIMR than intracraneal signals registered during non-epileptic seizures. In addition, EEG activity is more synchronized across areas during eyes closed than eyes open (e.g., Tan et al., 2013). Although there are not many studies comparing eyes closed and eyes open conditions in simultaneous frequencies or rhythms, it has been reported the CFC is higher in eyes closed than eyes open condition (Jirsa and Müller, 2013). Then, our hypothesis would be a higher value of MIMR in the eyes-closed than in the eyes-open condition.

**FIGURE 4:**
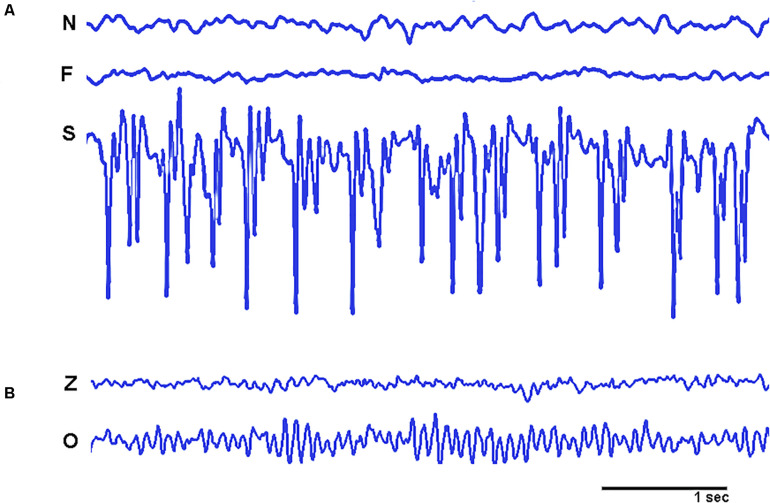
Graphical samples of the empirical signals used in the study. **(A)** Shows intracranial recordings from non-epileptic zone (N), Epileptic zone with non-ictal activity (F), and epileptic zone with ictal activity (S). **(B)** Shows EEG recordings from healthy participants from open eyes (Z) and closed eyes (O) conditions. Amplitudes of signals ranged from more than a hundred microvolts in intracranial recordings to a few microvolts in EEG signals.

We used two sets of data with 100 EEG segments each. These segments were collected from a database provided by Andrzejak et al. (2001). The first set of data was registered from 5 epilepsy patients using intracranial hippocampal recordings in three different conditions: Epileptic zone with non-ictal activity; Epileptic zone with non-ictal activity; and non-epileptic zone (see Figure 4 for a graphical sample). The length of each individual segment for all conditions (EEG and intracranial recordings) was 23.6 s, and it was digitized at A/D rate of 173.61 Hz and filtered with a band-pass of 0.53–40 Hz.

The second set was recorded from healthy participants resting in conditions of eyes open and eyes closed (see Figure 4 for a graphical sample). All EEG recordings were artifact free from 5 participants at scalp sites FP1, FP2, F7, F3, Fz, F4, F8, T3, C3, Cz, C4, T4, T5, P3, Pz, P4, T6, O1, and O2 (according with the 10–20 system).

In the process of MIMR calculation, we used window sizes of (WS: 9, 19, 27, 175) to obtain the binary sequences necessary for *Y(n).* These window sizes were selected to approximately capture classical rhythms of beta (^~^20 Hz), alpha (^~^10 Hz), theta (^~^6 Hz) and delta (≈1 Hz). The parameter τ was set to 15 in MIMR calculations for all empirical data.

### Statistical Analysis Description

**FIGURE 5:**
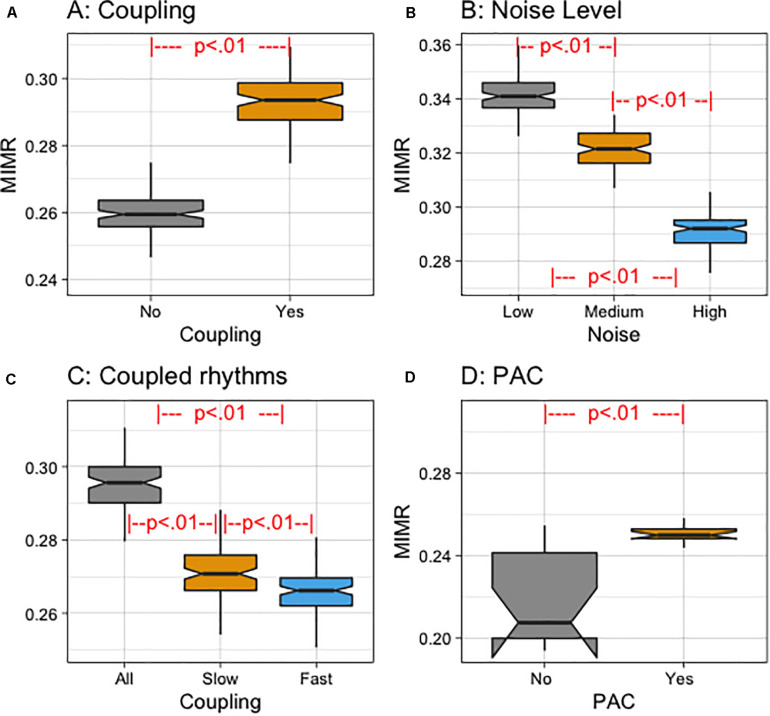
**(A)** Notched box plot of MIMR for different coupling conditions (Yes vs. No). **(B)** Notched box plot of MIMR for different noise levels (Low, Medium, and High). **(C)** Notched box plot of MIMR for different experimental conditions depending on the frequency of the rhythms that were coupled (All, Slow or Fast). **(D)** Notched box plot of MIMR for PAC and No PAC conditions of a signal with 8 and 60 Hz sinusoidal components.

As stated in the previous section, in order to validate MIMR, we compared different simulated signals at different time locking conditions, and empirical electric activity from the brain. After checking compliance with the parametric assumptions, we conducted mixed effects model ANOVAs. Random intercepts were included for participants. Analyses were performed in R (R Core Team, 2019) using the lmer() function of the lme4 package (Bates et al., 2014). When more than two conditions were involved in the comparison, pairwise comparisons were performed controlling for type I error increase with FDR algorithm (Benjamini and Hochberg, 1995) (all *p*–values were under 0.01). Regarding the simulated signals, we first compared signals with different time locking (see a summary of the results in Figure 5A), then we compared simulated signals with different noise levels (see a summary of the results in Figure 5B), and we manipulated the coupling of the wavelets in the signal (see a summary of the results in Figure 5C). Finally, we also generated synthetic signals in which we manipulated whether or not there was phase coupling (see a summary of the results in Figure 5D).

**FIGURE 6:**
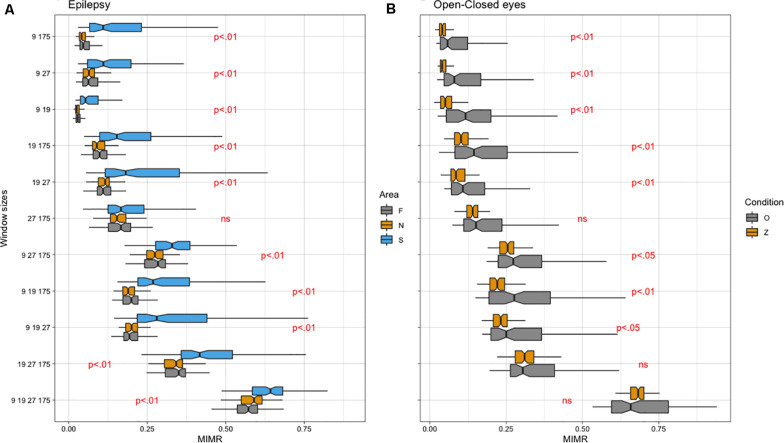
**(A)** Notched box plot of different areas (F, epileptic non-ictal, S: epileptic and N: non-epileptic) for each Window Size combination (WS). **(B)** Notched box plot of different conditions (O: closed eyes, Z: open eyes) for each WS. Note that WS (9, 19, 27, 175) approximately corresponds to frequencies of 19, 9, 6, and 1 Hz, respectively.

Regarding the empirical data, we first compared intracranial signals from the brain of patients with epilepsy. Then, we compared EEG signals from participants with their eyes open and closed. Due to the non-compliance of the parametric assumptions, we ran non-parametric ANOVAs (Friedman test). When significant differences were found and there were more than two conditions, we performed non-parametric pairwise comparisons (Wilcoxon test). The FDR procedure (Benjamini and Hochberg, 1995) was used to control the increase in type I errors due to the number of comparisons. Obtained results for empirical data are summarized in Figure 6.

## Results

### Results of Simulations

In the first instance we compared mean MIMR values when wavelet pulses were coupled or not coupled. We obtained three binary sequences from the simulated signal using window sizes for each frequency of the wavelets. As a result, each binary sequence reflected the activity at the scale of each wavelet. The window size for a particular frequency can be selected by taking into consideration the wavelength of the wavelet. This wavelength can be calculated by dividing the sample rate with the main frequency. In the calculation of MIMR for the simulated data, we used window sizes of (WS: 21, 33, 103) which were calculated to capture frequencies *f*_1_, *f*_2_, and *f*_3_, respectively. The time delay parameter τ we used in the estimation was set to 40; this value was approximated to the mean length at which the autocorrelation function showed the first minimum. Obtained results of coupling conditions (see Figure 5A) showed a significant effect of coupling on MIMR [*F*_(1, 98.99)_ = 1177.4, *p* < 0.01].

We then added three noise levels to the original signal (High, Medium and Low noise level). We applied MIMR to these compound signals and compared the mean results for each condition. Obtained results (see Figure 5B) showed significant differences between MIMR means [*F*_(2, 198)_ = 1444.9, *p* < 0.01].

We also manipulated the frequency of the rhythms that were coupled (All, only Slowest or only Fastest rhythms) (see section “Materials and Methods”). We then applied the MIMR on these signals and compared the obtained mean values. Obtained results (see Figure 5C) showed significant differences between means in both conditions [*F*_(2, 198)_ = 484.4, *p* < 0.01].

Finally, we compared signals composed of two frequencies (60 and 8 Hz), in which the amplitude of the fast component was totally determined by the phase of the slow one -condition: PAC-, with other signals, also composed of two frequencies (60 and 8 Hz), in which the amplitude of the fast component was modulated by a random factor condition: No PAC. Results (see Figure 5D) showed significant differences between means in both conditions [*F*_(1, 14)_ = 27.63, *p* < 0.01].

### Results of Empirical Data

#### Empirical Data From Patients With Epilepsy

We calculated the MIMR from different combinations of window sizes in the process of binarization (WS) applied to the original signals. The time delay parameter τ necessary for the calculation of MIMR was set to 15 (approximation of length in the series for a minimum in the autocorrelation function at all segments). We adjusted a linear model to the data with MIMR as the dependent variable and Area (F: epileptic, S: epileptic non-ictal and N: non-epileptic) as the within-participants factor. The results obtained with Friedman test indicated significant differences in MIMR between Areas for almost all window size combinations. See Figure 6 for a summary of the data and significant differences. Detailed quantitative information (statistics and *p*-values) can be found in Supplementary Table 1.

Regarding Pairwise comparisons, as can be seen (Figure 6A), MIMR discriminates well between different conditions, this discrimination being optimal for the most complex signal (WS: 9, 19, 27, 175). The only condition in which MIMR does not seem to show any difference was in the presence of the slowest rhythms (WS 27 175).

#### Open-Closed Eyes Empirical Conditions

We adjusted a linear model to the data with MIMR as the dependent variable and Condition (Z: Eyes open vs. O: eyes closed) as the between groups factor from different combinations of window size applied to the original signal. Obtained results for Wilcoxon test are summarized in Figure 6B and Supplementary Table 2. In general, the ability of MIMR to discriminate between conditions is very good, we did not find differences for some conditions that included slow rhythms (see Figure 6).

## Discussion

There is considerable experimental evidence showing that the coupling across neural oscillations may reflect integration processes within and across populations of neurons (Canolty and Knight, 2010; Fell and Axmacher, 2011; Xu et al., 2013). Several methods have been used to measure PAC. Although these methods have had some success, they have also faced numerous methodological challenges (Aru et al., 2015; van Wijk et al., 2015), such as the impact of the choice of bandwidth for band-pass filters, the consequences of the application of the Hilbert transformation on signals with closely spaced frequency components, short-time and weak disturbances, and the difficulty to detect cross-frequency coupling (Dupré la Tour et al., 2017).

In this paper we have presented and tested a novel strategy rooted in research on symbolic time-series analysis and based on Information Theory. We start from the idea of obtaining series of binary sequences based on the original EEG series in such a way that each of them includes information on a particular oscillatory activity. From these sequences, we have calculated the mutual delayed information and used it as an estimate of the dependence between them. This strategy has the advantage of not requiring phase or power calculation, and being applied to explore the coupling among several frequencies or oscillatory components.

In order to study the new measure, we applied it to simulated data and empirical EEG data. We carried out simulations in which we manipulated whether three wavelets were coupled or not. We found that the MIMR algorithm discriminated between both types of signals. In addition, the highest MIMR values were obtained for the complete coupling condition, where mutual information between the three wavelets was maximal. Furthermore, we have studied the robustness of the algorithm in the presence of 1/f noise. To do that, we generated three noise conditions (high, medium and low) and found significant differences in MIMR between the conditions. In addition, and consistent with predictions, in the high noise condition we obtained the smallest MIMR values while in the low noise condition we found the highest MIMR values. It is known that 1/f structure is not enough to simulate EEG series, there are specific temporal structures associated with oscillatory rhythms (He, 2014) present in this scale-free structure. The results we found using levels of 1/f noise indicated that MIMR is able to capture multiscale components coupled inside a general structure of 1/f noise. In addition, we also reinforced the validity of MIMR by including a set of simulations to test if MIMR was able to capture changes in PAC. In this case, we found that, when the coupling between the phase of a 8 Hz component was completely coupled with the amplitude of a 60 Hz component, MIMR exhibited lower values than when PAC was modulated by a small random component. Taken together, these simulations provide evidence indicating that MIMR might be useful to measure CFC of diverse nature.

Regarding MIMR behavior with the epilepsy empirical data, we found significant differences between experimental conditions (epileptic non-ictal, epileptic and non-epileptic) for most combinations of frequency bands. These results are consistent with current evidence about CFC in epilepsy. CFC has been used for example for seizure prediction (e.g., Alvarado-Rojas et al., 2014), and detection (Malladi et al., 2018). Edakawa et al. (2016) used PAC of intracranial EEG in 7 patients with temporal lobe epilepsy, and found high coupling between beta phase and gamma power during the ictal phase. Interestingly, the PAC measure was a better detector for ictal phases than gamma power alone (see also Weiss et al., 2013). But more importantly, we obtained higher MIMR values for those combinations that included more frequency bands. These results may indicate that during ictal conditions there is multifrequency coupling reflected in the statistical dependence between binary sequences from at least four rhythms.

A similar pattern of results was obtained for the open eye - closed-eye datasets. MIMR discriminated between the two conditions in almost all frequency band combinations. One study conducted by Barry et al. (2007) indicated that all frequency bands exhibited increased power values in the closed eye when compared with the open eye condition. In a different study, Gao et al. (2008), using non-linear detrended fluctuation analysis, found complexity changes at multiple scales. Taken together these results, they suggested that open eye vs. closed eye rhythms change at all scales and it could be a reflection of CFC. In this line, Jirsa and Müller (2013) found higher CFC in eyes closed than in eyes open conditions using CFC measures of power to power and power to frequency. Consistent with these results, we found that MIMR was, in general, reduced in the eyes open condition. However, we could not find statistical differences in conditions (WS 19 27 175) and (WS 9 19 27 175) which included slow rhythms. These results may indicate that slow rhythms are coupled in the same way in both conditions and, therefore, cortical long range patterns should be similar. Interestingly, this is also consistent with one of the findings from the study by Jirsa and Muller, in which they found different topographical patterns of CFC in eyes closed and eyes open conditions only at well localized cortical activity.

MIMR is a new measure that gives an additional perspective to the study of multiscale interactions in EEG signals; and because of that, we believe that it may be a good complement to other existing measures. For example, the advantage of PAC measures is that they explore an specific phase-amplitude relationship between two given frequencies. Although MIMR can be easily applied to two frequency bands by constructing two binary signals using the appropriate window sizes, the results are not so specific since they would reflect if the series from both frequencies show statistical time dependencies or couplings of any nature. This lack of specificity can be a limitation that needs to be taken into account. However, from our point of view, MIMR has two features that could make it a good candidate measure to explore the structure of EEG (or other physiological signals). The first one is that it does not need any assumption about the structure of the signal. This methodology can be applied to any linear or non-linear signal. Although the methodology provided by Martínez-Cancino et al. (2019) is also based in the calculation of mutual information, it requires phase-amplitude decomposition using the Hilbert transform and therefore it works on classical assumptions of linearity. In this context, our measure benefits to a greater extent from the use of information based methodology. The second advantage we would like to highlight is the possibility to explore various scales at the same time. This is a very important feature because it would be possible to explore if a given psychological function is better explained with the interaction of two specific rhythms or, on the contrary, can be explained as a result of the interactions between three or more rhythms. Furthermore, since mutual information is calculated on the symbolic series instead of the original series, as in symbolic analysis, efficiency of numerical computation is greatly increased (Piccardi, 2004). This is particularly interesting for all those applications that intend to use real-time MIMR (e.g., neurofeedback).

We are aware that, in this study, we present some data to validate a totally novel measure. More validation experiments are necessary and other variations of the same approach could be tested to improve the measure. Future work could aim to study more specific cognitive processes through EEGs, or to apply different methods of signal decomposition at different scales for a better understanding of the nature of the CFC in a given signal. In short, MIMR is a new measure that can be easily applied to neurophysiological signals, it does not take any *a priori* assumptions, and gives information about CFC by calculation of statistical dependence in time between two or more scales.

## Data Availability Statement

The datasets presented in this study can be found in online repositories. The names of the repository/repositories and accession number(s) can be found below: Synapse/syn22914762.

## Ethics Statement

In this work we do not use empirical data from our laboratory, so it has not been necessary to request permission from the Bioethics Committee of our university. The data used here are either simulated or from other laboratories, available upon request.

## Author Contributions

All authors listed have made a substantial, direct and intellectual contribution to the work, and approved it for publication.

## Conflict of Interest

The authors declare that the research was conducted in the absence of any commercial or financial relationships that could be construed as a potential conflict of interest.
