# Supplementary material for: Mutual Information of Multiple Rhythms for EEG Signals
Source: Front Neurosci. 2020 Dec 14;14:574796. doi: 10.3389/fnins.2020.574796 (PMC7768085; doi:10.3389/fnins.2020.574796)
Supplement: Supplementary file 1 [file Table_1.DOCX]

Supplementary Material

# Supplementary Tables

**Supplementary Table 1.** Friedman chi-squared values, associated *p*-values and Wilcoxon pairwise comparisons results for all window size combinations (WS)

| WS | Friedman chi-squared | *p*-value | Pairwise comparisons (Wilcoxon) |
| --- | --- | --- | --- |
| 9-19-27-175 | 57.62 | 3.076e-13 | F < N < S |
| 9-19-27 | 73.52 | 2.2e-16 | (F = N) < S |
| 19-27-175 | 44.24 | 2.474e-10 | (F = N) < S |
| 9-19-175 | 52.46 | 4.059e-12 | (F = N) < S |
| 9-27-175 | 30.78 | 2.071e-07 | (F = N) < S |
| 9-19 | 87.02 | 2.2e-16 | (F = N) < S |
| 9-27 | 36.02 | 1.508e-08 | (F = N) < S |
| 9-175 | 78.74 | 2.2e-16 | N < F < S |
| 27-175 | 3.66 | .1604 | ns |
| 19-27 | 37.52 | 7.123e-09 | (F = N) < S |
| 19-175 | 32.06 | 1.092e-07 | (F = N) < S |

**Supplementary Table 2.** Friedman chi-squared values, associated *p*-values and Wilcoxon pairwise comparisons results for all window size combinations (WS)

| WS | Wilcoxon statistic | *p*-value |
| --- | --- | --- |
| 9-19-27-175 | 4433 | .16 |
| 9-19-27 | 5834 | .04 |
| 19-27-175 | 5306 | .45 |
| 9-19-175 | 6493 | 2.65e-04 |
| 9-27-175 | 5952 | .02 |
| 9-19 | 7709 | 3.64e-11 |
| 9-27 | 7929 | 8.33e-13 |
| 9-175 | 6709 | 2.98e-05 |
| 27-175 | 5725 | .07 |
| 19- 27 | 6229 | 2.68e-3 |
| 19-175 | 6415 | 5.47e-4 |
